# Serum Irisin Predicts Posthepatectomy Complications in Patients with Hepatocellular Carcinoma

**DOI:** 10.1155/2019/9850191

**Published:** 2019-12-28

**Authors:** Jia Zhang, Mengyun Ke, Yifan Ren, Jianbin Bi, Zhaoqing Du, Mei Zhang, Yawen Wang, Lin Zhang, Zheng Wu, Yi Lv, Rongqian Wu

**Affiliations:** ^1^National Local Joint Engineering Research Center for Precision Surgery & Regenerative Medicine, Shaanxi Provincial Center for Regenerative Medicine and Surgical Engineering, First Affiliated Hospital of Xi'an Jiaotong University, Xi'an, Shaanxi Province, China; ^2^Department of Hepatobiliary Surgery, First Affiliated Hospital of Xi'an Jiaotong University, Xi'an, Shaanxi Province, China; ^3^Biobank, First Affiliated Hospital of Xi'an Jiaotong University, Xi'an, Shaanxi Province, China; ^4^Department of Laboratory Medicine, First Affiliated Hospital of Xi'an Jiaotong University, Xi'an, Shaanxi Province, China

## Abstract

**Background:**

Hepatectomy remains one of the most effective treatments for patients with hepatocellular carcinoma (HCC); however, it can lead to serious complications. Irisin, a key regulator of energy metabolism, is secreted into the circulation by shedding of the extracellular portion of the fibronectin type III domain-containing 5 (FNDC5). We have shown that irisin administration alleviates liver ischemia-reperfusion injury in mice. However, the role of preoperative irisin levels in HCC patients who underwent hepatectomy remained unknown. The purpose of this study was to determine how irisin expression changes in HCC and to explore the relationship between preoperative serum irisin levels and complications after hepatectomy.

**Methods:**

FNDC5/irisin expression data in HCC were extracted from The Cancer Genome Atlas (TCGA) dataset. A total of 219 participants, including 102 healthy controls and 117 HCC patients, were recruited in this study. All HCC patients underwent hepatectomy at the First Affiliated Hospital of the Xi'an Jiaotong University. Preoperative serum irisin levels were measured by ELISA. Postoperative complications were assessed using the comprehensive complication index (CCI) score. The Pearson rank correlation coefficient was computed to assess the correlation between preoperative serum irisin levels and postoperative CCI scores.

**Results:**

In TCGA dataset, FNDC5/irisin expression was downregulated in HCC tissues (*P* < 0.001). Similarly, serum irisin levels were decreased in HCC patients (*P* < 0.001). Low preoperative serum irisin levels were significantly correlated with high CCI scores after hepatectomy.

**Conclusions:**

Irisin may be a novel serum biomarker in the diagnosis of HCC and a predictor of complications after hepatectomy.

## 1. Introduction

Hepatocellular carcinoma (HCC) is a leading cause of cancer-related deaths worldwide. Hepatectomy remains one of the most effective treatments for patients with HCC; however, it can lead to serious complications. Irisin, a novel glycopeptide hormone, is secreted into the circulation by shedding of the extracellular portion of fibronectin type III domain-containing 5 (FNDC5) [1]. It was first identified in the skeletal muscles [1]. A recent comprehensive immunohistochemical study has shown that irisin is expressed in almost all human tissues [2]. Circulating irisin levels were decreased in breast cancer, and lower serum levels of irisin were associated with worse prognosis in breast cancer patients [3]. In cultured breast cancer cells, irisin reduced cell proliferation, viability, and migration and enhanced the cytotoxic effect of doxorubicin [4]. However, in HCC, one study showed that irisin expression was upregulated in HCC tissues [5], while another study did not [6]. These contradictory results indicate the complexity of the irisin expression/regulation in HCC.

Irisin is a key regulator of energy metabolism [7]. The liver plays a vital role in maintaining energy homeostasis including regulation of storage and release of energy. Our recent study has shown that irisin administration alleviates liver ischemia-reperfusion injury in mice [8]. However, the role of preoperative irisin levels in HCC patients who underwent hepatectomy remained unknown. The purpose of this study was to determine how irisin expression changes in HCC and to explore the relationship between preoperative serum irisin levels and complications after hepatectomy in HCC patients. We first analyzed HCC data of FNDC5/irisin expression in The Cancer Genome Atlas (TCGA) dataset, then measured circulating levels of irisin in HCC patients before liver resection, and investigated the relationship between preoperative serum irisin levels and complications after hepatectomy. The results would provide valuable information about FNDC5/irisin in HCC.

## 2. Materials and Methods

### 2.1. Patients

One hundred and seventeen patients with confirmed HCC who were diagnosed at the First Affiliated Hospital of the Xi'an Jiaotong University from 2012 to 2016 were included in this study. The diagnosis of HCC was based on typical imaging modalities by using contrast-enhanced computed tomography (CT), magnetic resonance image (MRI), angiography, and/or histopathology according to the American Association for the Study of Liver Diseases (AASLD) guideline. The clinicopathological data of patients with HCC at initial diagnosis were collected. TNM (tumor nodes metastasis) staging method was used. This study also included one hundred and two healthy volunteers as healthy controls. They were recruited from healthy volunteers who underwent routine physical examination at the First Affiliated Hospital of Xi'an Jiaotong University during the same period. The inclusion criteria for controls were the absence of cancer. The healthy controls were matched with the HCC patients by BMI (kg/m^2^, 23.5 ± 3.2 vs. 22.7 ± 2.8, *P* > 0.05), age (years, 53.6 ± 10.2 vs. 54.7 ± 11.1, *P* > 0.05), and gender (male/female, 82/20 vs. 94/23, *P* > 0.05). In this study, all experiments were approved by the Ethics Committee of the First Affiliated Hospital of Xi'an Jiaotong University (XJTU1AF2015LSL-057) and all patients gave their written informed consent before sample collection. All serum samples were stored at -80°C until analysis.

### 2.2. Measurement of Serum Irisin Levels

Serum irisin concentration was determined by enzyme-linked immunosorbent assay (ELISA) using a commercial kit (catalogue number: SEN576Hu, Cloud-Clone Corp USCN Life Science, Wuhan, China). The assay was conducted according to the manufacturer's instructions, and values were reported as *μ*g/ml. All specimens were tested blindly and in triplicate. The intra- and interassay variations were below 20%.

### 2.3. Assessment of Postoperative Complications

All 117 HCC patients in this cohort underwent curative hepatectomy at the First Affiliated Hospital of the Xi'an Jiaotong University. The postoperative complications were assessed using the comprehensive complication index (CCI) score [9]. The CCI was calculated as the sum of all postoperative complications that are weighted by their severity (available at https://www.assessurgery.com). Postoperative complications were defined as the occurrence of medical or surgical complications within 90 days of surgery. The severity of complications was evaluated using the Clavien-Dindo classification scale [10].

### 2.4. TCGA Data Extraction

FNDC5/irisin expression and clinicopathological parameters in HCC patients were downloaded from The Cancer Genome Atlas (TCGA; https://tcga-data.nci.nih.gov/tcga) data portal. The expression level of the FNDC5/irisin gene was compared between HCC tissues and noncancer tissues.

### 2.5. Statistical Analysis

Continuous data was tested for normality by the Kolmogorov-Smirnov test. For normal distribution variables, mean ± standard deviation (SD) was used for description and Student's *t*-test was used for comparison. For abnormal distribution variables, medians (interquartile range (IQR)) were used for description and Mann–Whitney rank-sum test was used for comparison. For categorical variables, absolute numbers and/or percent frequencies were used for description and Chi-square test or Fisher's exact test was used for comparison, as appropriate. Receiver operating characteristics (ROC) curves were generated to compare the diagnostic performance of serum irisin. SPSS software (version 18.0; SPSS Inc., Chicago, IL, United States) was used to detect statistical differences of groups, and two-tailed *P* value < 0.05 was accepted as significant.

## 3. Results

### 3.1. FNDC5/Irisin Expression Is Downregulated in HCC Tissue in TCGA Database

**Figure 1:**
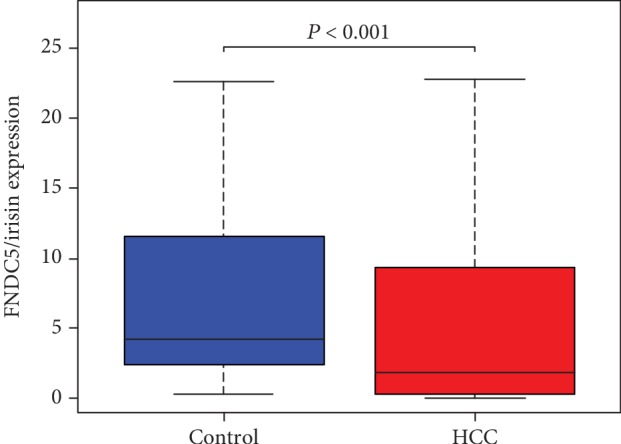
Hepatocellular carcinoma (HCC) data of FNDC5/irisin in The Cancer Genome Atlas (TCGA) database. FNDC5/irisin expression in HCC tissues (HCC) and noncancer tissues (non-cancer). Data are presented as medians (interquartile range (IQR)) and compared by the Mann–Whitney rank-sum test.

A total of 374 HCC cases and 50 non-HCC cases were included in TCGA database. As shown in Figure 1, FNDC5/irisin was downregulated in HCC tissues compared with noncancer tissues (*P* < 0.001).

### 3.2. Serum Irisin Levels Are Decreased in HCC Patients

**Figure 2:**
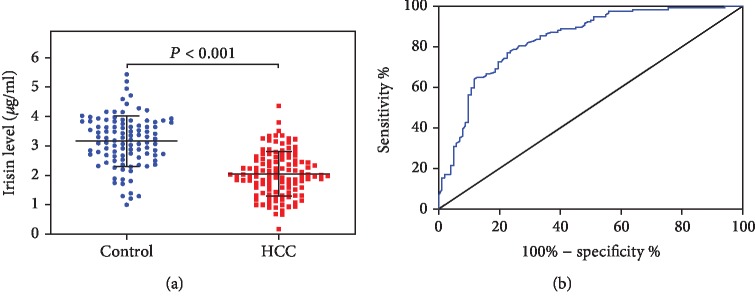
Serum irisin levels are decreased in hepatocellular carcinoma (HCC) patients. (a) Serum irisin levels in healthy controls and HCC patients. Serum irisin levels were measured by ELISA in healthy controls (Control, *n* = 102) and hepatocellular carcinoma patients (HCC, *n* = 117). Data were presented as the mean ± SD. Differences between the groups were compared by Student's *t*-test. (b) A receiver operating characteristic (ROC) curve of serum irisin to distinguish HCC patients from healthy controls.

A total of 219 participants, including 102 healthy controls and 117 HCC patients, were recruited in this study. As indicated in Figure 2(a), serum irisin levels in healthy controls and HCC patients were 3.16 ± 0.86 *μ*g/ml and 2.05 ± 0.83 *μ*g/ml, respectively. The difference was statistically significant (*P* < 0.001). The ROC analysis shows that the area under the ROC curve (AUC) of serum irisin to distinguish HCC patients from healthy controls was 0.8364 (95% CI 0.7826-0.8902, *P* < 0.0001, Figure 2(b)). The cut-off level of serum irisin was 2.62 *μ*g/ml. The sensitivity, specificity, and Youden index were 76.9%, 76.5%, and of 54.4%, respectively. The LR+, LR-, and diagnostic odd ratio were 3.3, 0.3, and 11.0, respectively.

### 3.3. Clinical Factors Associated with a Low Serum Irisin Level in HCC

Using the cut-off level of 2.62 *μ*g/ml, we identified 90 HCC patients in this cohort as having a low serum irisin level. As shown in Table 1, a low serum irisin level was associated with male, history of viral hepatitis, and a high AFP level (*P* < 0.05 for all).

### 3.4. Low Serum Irisin Levels Are Associated with High CCI Scores after Hepatectomy in HCC Patients

**Figure 3:**
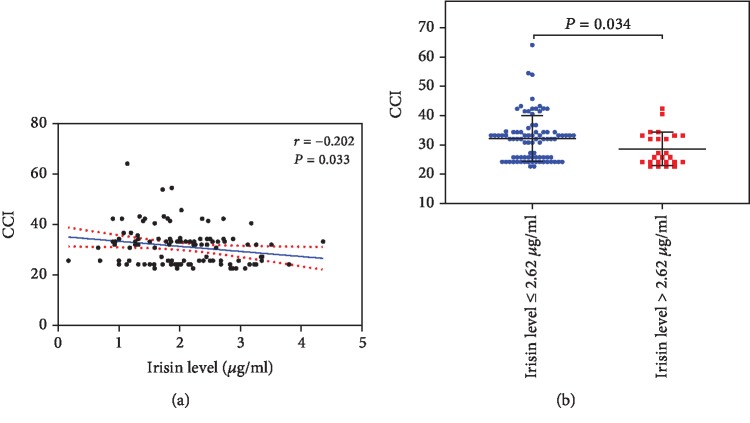
A low serum irisin level is associated with a high comprehensive complication index (CCI) score after hepatectomy in hepatocellular carcinoma (HCC) patients. All 117 HCC patients underwent curative hepatectomy in this cohort. (a) The Pearson rank correlation coefficient was computed to assess the correlation between preoperative serum irisin levels and postoperative CCI scores in HCC patients. (b) Postoperative CCI scores in HCC patients with serum irisin levels <2.62 *μ*g/ml (*n* = 90) or ≥2.62 *μ*g/ml (*n* = 27). Data were presented as the mean ± SD. Differences between the groups were compared by Student's *t*-test.

The median CCI score for the 117 HCC patients who underwent hepatectomy was 32.0 (range: 24.2-34.3). In order to determine whether preoperative irisin levels are associated with postoperative complications, we assessed the correlation between preoperative serum irisin levels and postoperative CCI scores in HCC patients. As shown in Figure 3(a), preoperative serum irisin levels were significantly correlated with postoperative CCI scores (*P* < 0.05). And HCC patients with a low preoperative serum irisin level (i.e., ≤2.62 *μ*g/ml) had significantly higher CCI scores after hepatectomy than those with a higher preoperative serum irisin level (*P* < 0.05, Figure 3(b)). Consequently, the patients with a lower preoperative serum irisin level had a longer hospital stay than those with a higher preoperative serum irisin level (*P* < 0.05, Table 2). Taken together, these results suggest the possibly use of serum irisin levels as a predictor for postoperative complications in HCC patients undergone hepatectomy.

## 4. Discussion

Irisin, named after the Greek messenger goddess Iris, was first described by Boström and colleagues in 2012 as a hormone that induces browning of subcutaneous fat [1]. Exercise increases the expression of peroxisome proliferator-activated receptor-*γ* coactivator-1*α* (PGC-1*α*) in the skeletal myocyte, which in turn drives the production of the membrane protein, FNDC5 [11]. FNDC5 is C-terminally cleaved and secreted as irisin into the circulation. The amino acid sequence of irisin is identical in humans and mice [12]. In TCGA data, FNDC5/irisin was significantly downregulated in HCC tissue. In the current study, we found that irisin was significantly decreased in the circulation of HCC patients and low preoperative serum irisin levels were significantly correlated with high CCI scores after hepatectomy. Thus, irisin may be a novel serum biomarker in the diagnosis of HCC and a predictor of serious complications after hepatectomy.

Recently, Gaggini et al. compared hepatic FNDC5/irisin gene expression in 18 patients with HCC undergoing liver transplantation and in 18 deceased liver donors [5]. They found that hepatic mRNA expression of FNDC5/irisin was higher in HCC patients than in donors. However, the sample size in that study was too small and they did not compare FNDC5/irisin expression between HCC tissues and adjacent noncancer tissues. Using irisin immunohistochemistry, Aydin et al. has showed that irisin expression was significantly increased in various gastrointestinal cancers, but not in HCC [6]. Irisin inhibits gluconeogenesis [13] and the liver plays key roles in the regulation of glucose production [14]. Cancer cells produce energy predominantly by a high rate of glycolysis. If irisin had been elevated in HCC, the energy reserves would have been depleted. Aydin believed this may be the reason why irisin levels do not increase in HCC [15]. In the current study, we found that irisin levels were deceased in HCC patients in both our cohort and TCGA cohort. Given the important role of irisin in energy metabolism, lower irisin expression in HCC may be attributed to an effort to control energy consumption and save the patient in the cachexia pathway.

In this study, we found that more male HCC patients had a serum irisin level below 2.62 *μ*g/ml, the cut-off level identified by the ROC analysis, than female HCC patients. This is not surprising. Anastasilakis et al. reported that resting irisin levels were significantly higher in healthy young females than in healthy young males [16]. And in response to acute aerobic exercise, irisin levels increased significantly more in lean women than in lean men [17]. Thus, the difference in irisin levels between male and female HCC patients may simply reflect the difference between male and female in the general population. A low serum irisin was also associated with history of viral hepatitis. To our knowledge, the role of irisin has not been studied in viral hepatitis. In our cohort, the majority of HCC patients had viral hepatitis background. Our result suggests that this area merits further investigation. AFP remains the most extensively used serum biomarker for the diagnosis of HCC patients. Our result shows that the level of AFP in the low serum irisin group was more than 14 times of that in the high serum irisin group. Thus, irisin may be used side by side with AFP for the diagnosis of HCC.

Despite recent advances in operative techniques and perioperative care, major liver resection is still associated with relatively high incidences of postoperative complications. Thus, the preoperative assessment of risk factors for serious postoperative complications is critical. A recent meta-analysis and systematic review indicated that preoperative exercise such as inspiratory muscle training, aerobic exercise, and/or resistance training can reduce postoperative complications after abdominal surgery [18]. In addition, regular physical activity is known to lower the risk of developing cancer [19]. The benefits of exercise continue after diagnosis as it improves overall quality of life in cancer survivors. Acute and intensive exercise increases irisin's release into the circulation [14]. Our recent study has shown that administration of exogenous irisin alleviates liver ischemia-reperfusion injury by inhibiting excessive mitochondrial fission, promoting mitochondrial biogenesis, and decreasing oxidative stress [8]. In the current study, we found that low preoperative serum irisin levels were significantly correlated with high CCI scores after hepatectomy. The CCI score was recently developed to evaluate the summative severity of all major and minor postoperative [9]. Thus, the beneficial effects of preoperative exercise on outcomes after surgery may be related to the increased level of circulating irisin in these patients and the preoperative serum irisin level can be used to evaluate risks of overall postoperative complications.

In the present study, we found that serum irisin levels were at a few *μ*g/ml. However, circulating irisin levels have been reported at *μ*g/ml [3, 20], ng/ml [21, 22], and even pg/ml [23, 24] levels in different studies. In most studies, irisin levels were measured by ELISA. The discrepancies in irisin levels observed may reflect differences in primary antibodies, interpopulation or methodological variations, assay discrepancies, and/or preanalytical variability (i.e., blood collection, handling, storage, and repeated melting). However, the exact reason remains unknown [25]. In this regard, we recommend to use the same assay kit and to measure all the samples in a study at the same time.

There are some limitations in the current study. The healthy controls were recruited from healthy volunteers who underwent routine physical examination at our hospital during the same period. Since many biochemical indicators for HCC patients are not included in the routine physical examination, we were unable to present them here. However, we believe the data presented in the current manuscript are sufficient to prove that serum irisin levels were decreased in HCC patients and low preoperative serum irisin levels were a predictor of complications after hepatectomy. Thus, the absence of biochemical indicators of healthy controls did not affect our main conclusion. However, it would be interesting to investigate how serum levels of irisin correlate with other biochemical indicators of HCC in the future. In addition, how do irisin levels change after hepatectomy also warrants further investigation.

## 5. Conclusion

FNDC5/irisin expression was significantly downregulated in patients with HCC. Serum irisin may be a novel biomarker in the diagnosis of HCC, and low preoperative serum irisin levels were significantly correlated with high CCI scores after hepatectomy. The potential role of FNDC5/irisin in the development and progression of HCC would be an interesting area for future investigation.

## Figures and Tables

**Table 1:** Demographic data, coexistences, laboratory results, and imaging findings in HCC patients.

Variable	Total (*N* = 117)	Irisin ≥ 2.62 *μ*g/ml (*N* = 27)	Irisin < 2.62 *μ*g/ml (*N* = 90)	*P* value
Age (years, mean ± SD)	54.7 ± 11.1	54.4 ± 13.3	54.8 ± 10.5	0.905
Gender (male/female)	94/23	18/9	76/14	**0.041**
BMI (kg/m^2^, mean ± SD)	22.7 ± 2.8	23.1 ± 2.8	22.6 ± 2.8	0.468
Coexistences (*n*, %)				
Smoking	59 (50.4)	13 (48.1)	46 (51.1)	0.787
Drinking	40 (34.2)	9 (33.3)	31 (34.4)	0.915
Hypertension	24 (20.5)	8 (29.6)	16 (17.8)	0.181
Diabetes mellitus	10 (8.5)	2 (7.4)	8 (8.9)	0.809
Virus hepatitis	86 (73.5)	13 (48.1)	73 (81.1)	**0.001**
Cirrhosis	62 (53.0)	12 (44.4)	50 (55.6)	0.310
Laboratory results (mean ± SD or median, interquartile range)				
AFP (ng/ml)	111.2 (4.7-106.8)	11.2 (3.0-295.5)	166.3 (5.3-2616.8)	**0.030**
Leucocytes (×10^9^/l)	5.3 ± 2.4	5.6 ± 2.7	5.3 ± 2.4	0.477
Hemoglobin (g/l)	134.9 ± 20.9	132.9 ± 23.0	135.4 ± 20.3	0.580
Platelet count (×10^9^/l)	145.1 ± 64.7	157.4 ± 59.0	141.5 ± 66.2	0.263
ALT (U/l)	39.3 (22.5-62.9)	38.0 (22.8-71.1)	40.0 (22.1-60.1)	0.313
AST (U/l)	38.0 (25.3-58.3)	37.9 (25.0-60.2)	38.3 (25.8-58.4)	0.779
ALP (U/l)	101.8 (73.8-132.8)	102.9 (71.0-124.0)	102.0 (74.6-136.0)	0.876
GGT (U/l)	73.5 (41.6-136.8)	66.0 (45.6-135.32)	79.4 (39.2-140.5)	0.954
TBIL (*μ*mol/l)	17.4 ± 11.0	15.5 ± 6.2	18.0 ± 12.1	0.932
DBIL (*μ*mol/l)	7.7 ± 8.3	6.5 ± 4.9	8.0 ± 9.1	0.422
ALB (g/l)	37.5 ± 4.7	37.4 ± 6.2	37.6 ± 4.1	0.916
Cr (*μ*mol/l)	60.8 ± 13.7	64.7 ± 17.7	59.7 ± 12.1	0.093
BUN (mmol/l)	5.1 ± 1.5	5.1 ± 1.7	5.1 ± 1.5	0.804
PT (s)	14.0 ± 1.3	13.7 ± 1.3	14.1 ± 1.3	0.112
INR	1.11 ± 0.13	1.06 ± 0.13	1.12 ± 0.13	0.063
Imaging findings (mean ± SD or *n*)				
Tumor size (cm)	6.9 ± 3.6	6.0 ± 3.0	7.2 ± 3.7	0.137
Single/multiple tumor	94/23	21/6	73/17	0.702
TNM stage (I, II/III, IV)	48/69	12/15	35/55	0.606

BMI: body mass index; AFP: alpha-fetoprotein; ALT: alanine transaminase; AST: aspartate transaminase; ALP: alkaline phosphatase; GGT: gamma-glutamyl transpeptidase; TBIL: total bilirubin; DBIL: direct bilirubin; ALB: albumin; Cr: creatinine; BUN: blood urea nitrogen; PT: prothrombin time; INR: international normalized ratio.

**Table 2:** Comprehensive complication index (CCI), complications, and outcomes in HCC patients.

Variable (mean ± SD or *n* (%))	Total (*N* = 117)	Irisin ≥ 2.62 *μ*g/ml (*N* = 27)	Irisin < 2.62 *μ*g/ml (*N* = 90)	*P* value
Comprehensive complication index (CCI)	31.5 ± 7.8	28.6 ± 5.7	32.3 ± 8.2	**0.021**
Complications				
Peritoneal effusion	27 (23.1%)	6 (22.2%)	21 (23.3%)	0.904
Intra-abdominal hemorrhage	2 (1.7%)	1 (3.7%)	1 (1.1%)	0.410
Infectious complications	20 (17.1%)	3 (11.1%)	17 (18.9%)	0.516
Pulmonary infection	15 (12.8%)	3 (11.1%)	12 (13.3%)	1
Hepatic abscess	3 (2.6%)	0 (0)	3 (3.3%)	1
Sepsis	1 (0.9%)	0 (0)	1 (1.1%)	1
Other infections	1 (0.9%)	0 (0)	1 (1.1%)	1
Biliary complications	6 (5.1%)	0 (0)	6 (6.7%)	0.334
Bile leakage	5 (4.3%)	0 (0)	5 (5.6%)	0.478
Biliary stricture	1 (0.9%)	0 (0)	1 (1.1%)	1
Organ failure	5 (4.3%)	1 (3.7%)	4 (4.4%)	1
Renal failure	3 (2.6%)	0 (0)	3 (3.3%)	1
Liver failure	1 (0.9%)	0 (0)	1 (1.1%)	1
MODS	1 (0.9%)	1 (3.7)	0 (0)	0.231
Outcomes				
Length of ICU stay	1.6 ± 1.2	1.3 ± 0.8	1.6 ± 1.3	0.593
Length of hospital stay (days)	19.4 ± 6.9	16.9 ± 5.3	20.1 ± 7.2	**0.041**
Mortality in 3 years	48 (41.0%)	13 (48.1%)	35 (38.9%)	0.391

## Data Availability

The data used to support the findings of this study are included within the article.

## Abbreviations

BMI: Body mass index
AFP: Alpha-fetoprotein
ALT: Alanine transaminase
AST: Aspartate transaminase
ALP: Alkaline phosphatase
GGT: Gamma-glutamyl transpeptidase
TBIL: Total bilirubin
DBIL: Direct bilirubin
ALB: Albumin
Cr: Creatinine
BUN: Blood urea nitrogen
PT: Prothrombin time
INR: International normalized ratio
HCC: Hepatocellular carcinoma
FNDC5: Fibronectin type III domain-containing 5
TCGA: The Cancer Genome Atlas
CCI: Comprehensive complication index
CT: Computed tomography
TNM: Tumor nodes metastasis.
